# Supplementary material for: Causal Relationship between Aging and Anorexia Nervosa: A White-Matter-Microstructure-Mediated Mendelian Randomization Analysis
Source: Biomedicines. 2024 Aug 16;12(8):1874. doi: 10.3390/biomedicines12081874 (PMC11351342; doi:10.3390/biomedicines12081874)
Supplement: Supplementary file 1 [file biomedicines-12-01874-s001.zip › Supplementary Figure/Figure S4/Singe SNP plot of MR analysis for TL to ubm-b-2001.pdf]

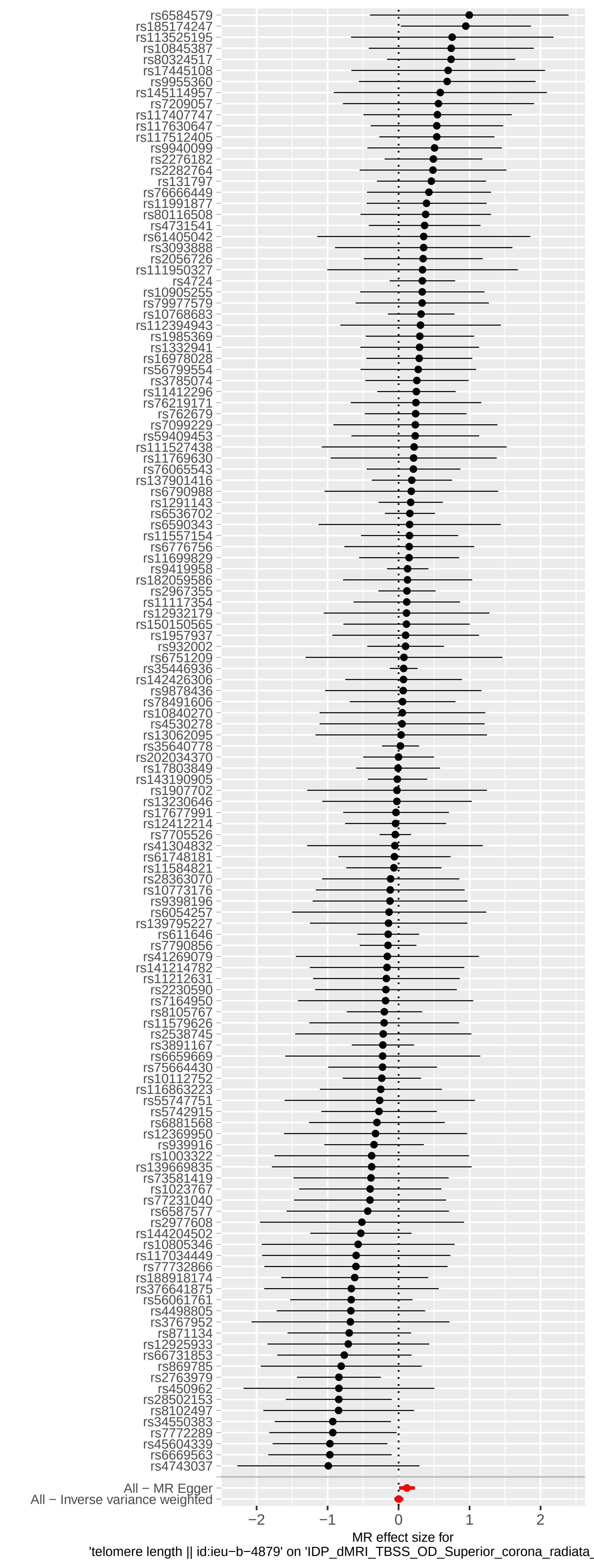
rs6584579
rs185174247
rs113525195
rs10845387
rs80324517
rs17445108
rs9955360
rs145114957
rs7209057
rs117407747
rs117630647
rs117512405
rs9940099
rs2276182
rs2282764
rs131797
rs76666449
rs11991877
rs80116508
rs4731541
rs61405042
rs3093888
rs2056726
rs111950327
rs4724
rs10905255
rs79977579
rs10768683
rs112394943
rs1985369
rs1332941
rs16978028
rs56799554
rs3785074
rs11412296
rs76219171
rs762679
rs7099229
rs59409453
rs111527438
rs11769630
rs76065543
rs137901416
rs6790988
rs1291143
rs6536702
rs6590343
rs11557154
rs6776756
rs11699829
rs9419958
rs182059586
rs2967355
rs11117354
rs12932179
rs150150565
rs1957937
rs932002
rs6751209
rs35446936
rs142426306
rs9878436
rs78491606
rs10840270
rs4530278
rs13062095
rs35640778
rs202034370
rs17803849
rs143190905
rs1907702
rs13230646
rs17677991
rs12412214
rs7705526
rs41304832
rs61748181
rs11584821
rs28363070
rs10773176
rs9398196
rs6054257
rs139795227
rs611646
rs7790856
rs41269079
rs141214782
rs11212631
rs2230590
rs7164950
rs8105767
rs11579626
rs2538745
rs3891167
rs6659669
rs75664430
rs10112752
rs116863223
rs55747751
rs5742915
rs6881568
rs12369950
rs939916
rs1003322
rs139669835
rs73581419
rs1023767
rs77231040
rs6587577
rs2977608
rs144204502
rs10805346
rs117034449
rs77732866
rs188918174
rs376641875
rs56061761
rs4498805
rs3767952
rs871134
rs12925933
rs66731853
rs869785
rs2763979
rs450962
rs28502153
rs8102497
rs34550383
rs7772289
rs45604339
rs6669563
rs4743037
All − MR Egger
All − Inverse variance weighted
−2
−1
0
1
2
MR effect size for
'telomere length || id:ieu−b−4879' on 'IDP_dMRI_TBSS_OD_Superior_corona_radiata_
